# Exploratory investigation of region level risk factors of Ebola Virus Disease in West Africa

**DOI:** 10.7717/peerj.5888

**Published:** 2018-11-19

**Authors:** Benjamin Levy, Agricola Odoi

**Affiliations:** 1 Department of Mathematics, Fitchburg State University, Fitchburg, MA, United States of America; 2 Department of Biomedical and Diagnostic Sciences, University of Tennessee—Knoxville, Knoxville, TN, United States of America

**Keywords:** Ebola, West Africa, Negative binomial regression, Poisson regression, Statistical modeling, Epidemiology

## Abstract

**Background:**

Ebola Virus Disease (EVD) is a highly infectious disease that has produced over 25,000 cases in the past 50 years. While many past outbreaks resulted in relatively few cases, the 2014 outbreak in West Africa was the most deadly occurrence of EVD to date, producing over 15,000 confirmed cases.

**Objective:**

In this study, we investigated population level predictors of EVD risk at the regional level in Sierra Leone, Liberia, and Guinea.

**Methods:**

Spatial and descriptive analyses were conducted to assess distribution of EVD cases. Choropleth maps showing the spatial distribution of EVD risk across the study area were generated in ArcGIS. Poisson and negative binomial models were then used to investigate population and regional predictors of EVD risk.

**Results:**

Results indicated that the risk of EVD was significantly lower in areas with higher proportions of: (a) the population living in urban areas, (b) households with a low quality or no toilets, and (c) married men working in blue collar jobs. However, risk of EVD was significantly higher in areas with high mean years of education.

**Conclusions:**

The identified significant predictors of high risk were associated with areas with higher levels of urbanization. This may be due to higher population densities in the more urban centers and hence higher potential of infectious contact. However, there is need to better understand the role of urbanization and individual contact structure in an Ebola outbreak. We discuss shortcomings in available data and emphasize the need to consider spatial scale in future data collection and epidemiological studies.

## Introduction

Ebola Virus Disease (EVD) is endemic to Africa and poses a significant public health threat in the region. The virus is transmitted by direct contact with bodily fluids of an infected individual. The disease has an incubation period of 2 to 21 days (WHO Ebola Response Team, 2014; World Health Organization, 2015). Early symptoms include fever, fatigue, muscle pain, headache, and sore throat that later develop into vomiting, diarrhea, rash, impaired kidney and liver function, as well as internal and external bleeding (WHO Ebola Response Team, 2014; World Health Organization, 2015). Since many of the early symptoms are similar to those of other diseases such as influenza, malaria, and typhoid fever, diagnosis of EVD is challenging without detailed blood work. Supportive therapy typically involves re-hydration with oral or intravenous fluids as well as addressing other specific symptoms (World Health Organization, 2015). The recombinant vesicular stomatitis virus-Zaire Ebola virus vaccine (rVSV-ZEBOV) was developed shortly after the outbreak in West Africa and studies have shown that it provides significant protection (Henao-Restrepo et  al., 2015).

Since the initial outbreak of EVD in Sudan in 1976, there have been 21 outbreaks in Africa resulting in over 25,000 cases (Center for Disease Control, 2014; Reza et al., 2015). The highly infectious disease also has a staggering case fatality rate with approximately 60% of all historical cases ending in death (Center for Disease Control, 2014). The 2014 West African outbreak began in Guinea in March 2014 and spread to the neighboring countries of Liberia and Sierra Leone (Baize et al., 2014). While the disease spread throughout these three underprepared countries, contact tracing and isolation techniques prevented the disease from spreading to other countries.

There has been a concerted effort by the scientific community to learn from the 2014 EVD outbreak. Bats are believed to be reservoirs for the virus, but it is possible that other animals in the region also harbor the disease. To determine the risks and behaviors associated with zoonotic transmission of the disease, several studies have investigated how humans in West Africa interact with the environment (Fang et al., 2016; Richards et al., 2015; Walsh & Haseeb, 2015). A number of studies have also analyzed how the disease was transmitted among humans, some of which considered the types of contacts associated with EVD transmission at the individual level (Agua-Agum et al., 2016; Brainard et al., 2016; Francesconi et al., 2003; Lau et al., 2017; Richards et al., 2015), while others investigated the characteristics of the within-host progression of the disease (Haaskjold et al., 2016; Reza et al., 2015). Many studies were also conducted at the population level (Agua-Agum et al., 2016; Fang et al., 2016; Francesconi et al., 2003; Haaskjold et al., 2016; Lau et al., 2017; Moyen et al., 2015; Valeri et al., 2016; Walsh & Haseeb, 2015). Some findings agree on risk factors for contracting and dying from the disease as well as the likely presence of “superspreaders” in the population, while others have illustrated the need for a better understanding of population level spread of the disease (Lau et al., 2017; Richards et al., 2015; Valeri et al., 2016; Walsh & Haseeb, 2015). In this study, we investigated region level predictors of EVD to help guide future studies and disease control efforts.

## Methods

### Study area and data sources

**Figure 1:**
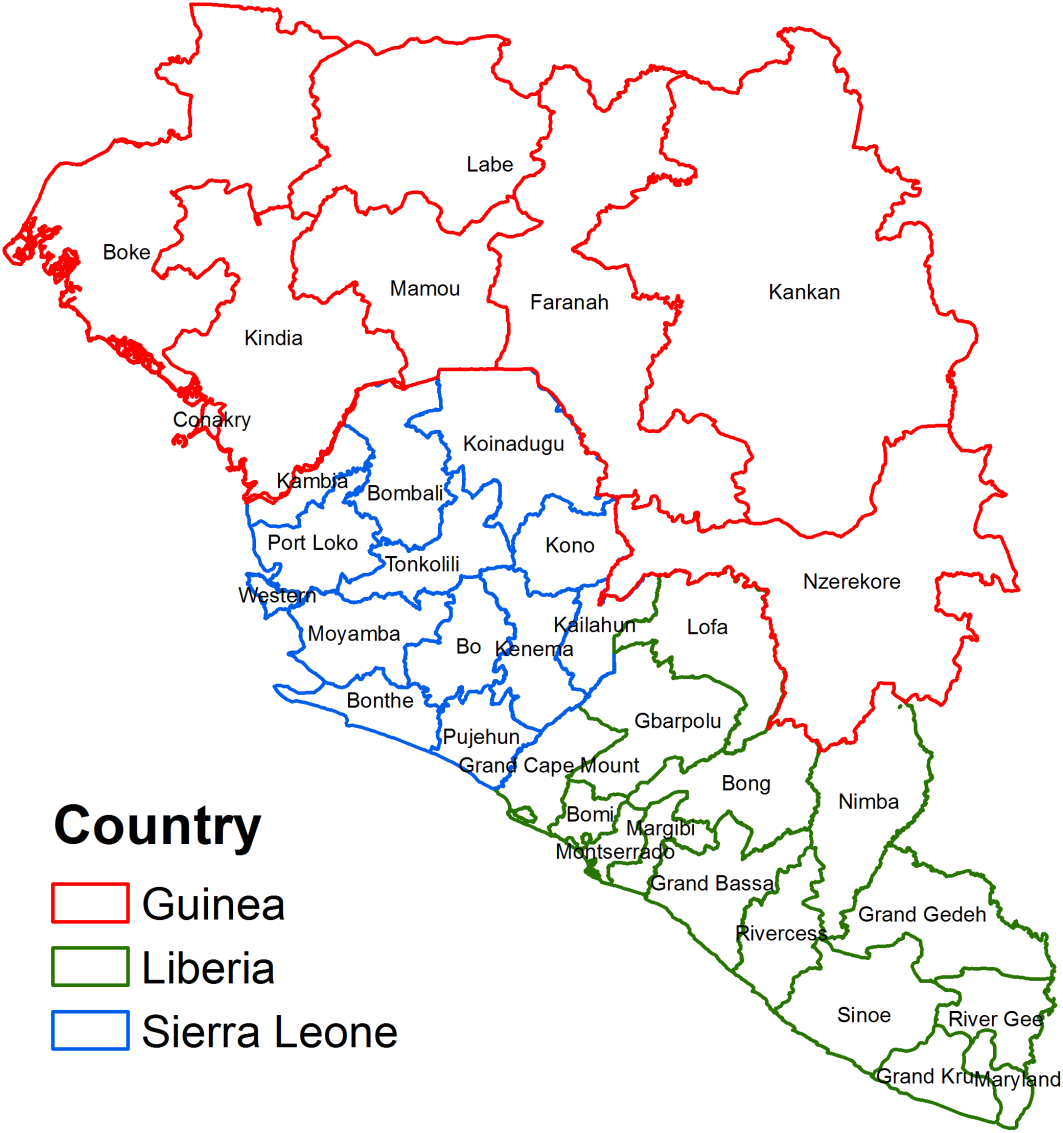
Map of the study area showing regions in West African countries affected by the 2014 Ebola Virus Disease outbreak.

The study area, with a population of 20,184,666 people, consisted of regions in countries that were most affected by the 2014 EVD outbreak: Sierra Leone, Guinea, and Liberia (Fig. 1). The dependent variable in the study was the number of confirmed cases of Ebola for each region; this information was obtained from the World Health Organization (World Health Organization, 2016). Confirmed cases were defined as those that were confirmed to be positive by a laboratory using one of the available diagnostic tests. Diagnostic tests used during the West African outbreak varied depending on the time since infection, health status of the individual, and resources available. Within a few days of onset of symptoms, a polymerase chain reaction (PCR), virus isolation, or an ELISA test would be used. Late in the disease course or after recovery an IgM ELISA test or IgG antibody test was used. For deceased patients, a PCR test, immunohistochemistry test, or virus isolation was used (Center for Disease Control, 2015).

Table 1 lists all variables assessed for potential association with risk of Ebola Disease. These data were compiled by Global Data Lab (GDL) which supplied 20 potential predictors at the regional level from demographic and health surveys conducted in the affected countries (Global Data Labs, 2014). Thus, predictors considered for assessment included average level of education of persons aged 20–49, percentage of population living in an urban setting, percentage of households with electricity, and of households with either no toilet or a toilet that lacks plumbing, among others (Liberia Institute of Statistics and Geo-Information Services, LISGIS)et al.(2014; Statistics Sierra Leone (SSL) & ICF International, 2014; Ministry of Health and Public Hygiene (Guinea), 2013). Global Data Lab also derived a Mean International Wealth Index score for each region, which is a measure of the average household’s relative wealth (Smits & Steendijk, 2015). Finally, the population density of each region was obtained from three sources (Institute National de la Statistique, 2015; Liberia Institute of Statistics and Geo-Information Services , LISGIS; NetHope Open Humanitarian Data Repository, 2016).

**Table 1:** Variables investigated as potential predictors of Ebola Virus Disease risk.

**Variable**	**Min**	**Max**	**Mean**	**Standard deviation**
Wealth index	15.3	72.6	26.3	11
Mean years of education of persons 20–49	1.5	8.2	3.7	1.7
Mean years of education of women 20–49	0.7	6.9	2.4	1.4
Mean years of education of men 20–49	2.4	9.5	5	1.9
Percent of population living in urban area	4.4	100	28.7	24.7
Percent of married men age 20–49 working in agriculture	3.7	84	56.5	19.1
Percent of married men age 20–49 working in blue collar jobs	10.5	73.2	36	15.8
Percent of married men age 20–49 working in white collar jobs	1.8	23.1	7.5	4.4
Percent of households with a television	0.7	85.2	11.7	16.9
Percent of households with a telephone	28.8	97.6	57.1	15.3
Percent of households with electricity	0	94	9.6	18.3
Percent of households with 0–1 rooms designated for sleeping	4.5	35.8	15.5	7.4
Percent of households with >3 rooms designated for sleeping	30.6	77	57.8	11.5
Percent of households with a high quality floor	0.4	33	4.2	6.86
Percent of households with a low quality floor	5.1	92.4	60.7	20.1
Percent of households with piped water	0	84.7	5.3	14.9
Percent of households with bad quality water supply	0.9	79.1	38.1	20.5
Percent of households with a flush toilet	0.1	62.5	7.4	13.1
Percent of households with bad quality or no toilet	8.9	89.6	61.5	17.3
Population density	8.4	3,706.4	245.2	776.6

### Investigation of predictors of EVD

The data were compiled into a single Microsoft Excel file. The data were then assessed for inconsistencies, summary statistics computed, and spatial analyses performed. The summary statistics were computed for the entire dataset as well as for each of the three countries separately. To investigate predictors of EVD, two types of statistical models were fitted to the data: Poisson and negative binomial models. All statistical analyses were performed in STATA statistical software version 14 (StatCorp, Tulsa, OK, USA).

#### Univariable and multivariable Poisson and negative binomial models

Initial investigation of predictors of EVD risk involved use of Poisson models. This involved first assessing univariable associations between the outcome (region level numbers of cases of EVD) with each of the predictors using a liberal *p* −value of 0.15. We used the log of the population estimates as an offset. The exponentials of the regression coefficients of the resulting models yielded risk ratios (relative risks) as measures of association. The resulting unadjusted risk ratios and *p*-values produced from each of the univariable models were noted and recorded. Since all predictors assessed using the univariable Poisson models produced *p* −values less than 0.15, we used a stepwise backwards elimination model building strategy to identify significant predictors in a multivariable Poisson model using a more strict *p* −value <0.05 for entry and retention in the model. The stepwise backwards elimination process began with all predictors in the model and removed statistically non-significant variables one at a time until all predictors in the model had *p* < 0.05. Overall goodness-of-fit of the final Poisson model was assessed using Deviance Chi-square.

A key characteristic of Poisson models is that the mean and variance of the data are assumed to be equal, which implies that the degrees of freedom (*df*) be equal to the deviance so that $ \frac{1}{df} \mathrm{Deviance}\approx 1$ (Dohoo, Martin & Stryhn, 2003). The final model showed strong evidence of overdispersion producing $ \frac{1}{df} \mathrm{Deviance}\gt 100$ as the test for overdispersion was highly significant (*p* < .001). The presence of overdispersion in the data indicates that the variance is larger than the mean and therefore a Poisson model is not appropriate for these data. Unlike Poisson models, negative binomial models assume that the variance exceeds the mean by a factor (*α*), which depends on the mean (Dohoo, Martin & Stryhn, 2003). As a result, a negative binomial model was deemed to be a better choice given our data and was used in all subsequent analyses.

A negative binomial model was fit to the data again using the log of the population estimates as an offset. The exponentials of the regression coefficients of these models yielded adjusted risk ratios as the measures of association. As with the Poisson model, we first assessed each predictor for simple associations with the outcome using univariable negative binomial models. Unadjusted risk ratios and *p*-values were also computed to assess potential univariable associations between the potential predictor and outcome. Then, a stepwise backwards eliminations procedure was used to identify statistically significant predictors of EVD in a multivariable negative binomial model. The same procedure used in the Poisson model was used again here with a threshold of *p* ≥ 0.05 for removal from the model. To assess potential confounding, we calculated the percent change in regression coefficients with the suspected confounding variable included in the model versus when it was not included. We considered any percent change exceeding 20% as an indication of confounding. Since population density had previously been shown to be a significant predictor of EVD but was not significant in any of our models, we used the aforementioned process to check if it was an important confounder in our multivariable models. Assessment of multicollinearity showed no significant collinearity between the predictors in the final model. Finally, two-way interaction terms of all significant variables were assessed. Both linear and nonlinear combinations were tested and no interactions were found to be significant. Adjusted risk ratios and *p*-values for each of the significant predictors in the final negative binomial model were computed and recorded. Choropleth maps of the geographic distributions of the outcome and predictors that were significantly associated with the outcome in the final negative binomial model were generated in ArcGIS 10.1.

## Results

### Summary statistics

Table 2 shows the population, total numbers of confirmed cases, and risk of EVD in each region. The population values range as low as 57,913 individuals in Grand Kru, Liberia to as high as 1,986,329 in Kankan, Guinea. Moreover, there were noticeable variations in the numbers of confirmed cases, the values of which are not directly related to the population in a given region. For instance, the number of confirmed cases of EVD in the seven regions with a population over one million people ranged from 80 (one of the lowest values in the dataset) to 3,449 confirmed cases (the largest value in the dataset). The risk values also reflect this trend ranging from as few as one case per 100,000 individuals in the populous region of Labe, Guinea, to as many as 296 cases per 100,000 in the moderately populated Port Loko, Sierra Leone. Table 3 displays population, number of confirmed cases, and Ebola disease risk for each country (Liberia Institute of Statistics and Geo-Information Services (LISGIS) et al., 2008; Republic of Sierra Leone, 2010; Guinea Ministry of Planning, 2014). Although Guinea has the highest population, it has the lowest EVD risk. Sierra Leone, on the other hand, has both the highest number of confirmed cases of EVD and the highest EVD risk.

**Table 2:** A list of regions, their corresponding population estimates, and the total confirmed number of cases of Ebola Virus Disease.

**Country**	**Region**	**Population**	**Confirmed cases of Ebola**	**Risk (cases/100,000)**
Guinea	Boke	1,081,445	80	7
Guinea	Conakry	1,667,864	568	34
Guinea	Faranah	942,733	154	16
Guinea	Kankan	1,986,329	235	12
Guinea	Kindia	1,559,185	924	59
Guinea	Labe	995,717	7	1
Guinea	Mamou	732,117	16	2
Guinea	Nzerekore	1,663,582	1,351	81
Liberia	Bomi	84,119	139	165
Liberia	Bong	333,481	150	45
Liberia	Gbarpolu	83,388	16	19
Liberia	Grand Bassa	221,693	54	24
Liberia	Grand Cape Mount	127,076	94	74
Liberia	Grand Gedeh	125,258	3	2
Liberia	Grand Kru	57,913	4	7
Liberia	Lofa	276,863	332	120
Liberia	Margibi	209,923	392	187
Liberia	Maryland	135,938	4	3
Liberia	Montserrado	1,118,241	1,797	161
Liberia	Nimba	462,026	116	25
Liberia	River Cess	71,509	24	34
Liberia	River Gee	66,789	8	12
Liberia	Sinoe	102,391	18	18
Sierra Leone	Bo	561,524	314	56
Sierra Leone	Bombali	434,319	1,049	242
Sierra Leone	Bonthe	140,845	5	4
Sierra Leone	Kailahun	409,520	565	138
Sierra Leone	Kambia	313,765	253	81
Sierra Leone	Kenema	545,327	503	92
Sierra Leone	Koinadugu	251,091	109	43
Sierra Leone	Kono	352,328	254	72
Sierra Leone	Moyamba	252,390	209	83
Sierra Leone	Port Loko	500,992	1,485	296
Sierra Leone	Pujehun	252,390	31	12
Sierra Leone	Tonkolili	385,322	457	119
Sierra Leone	Western	1,679,273	3,449	205
**Total**		**20,184,666**	**15,169**	**75**

A look at the summary statistics for all three countries combined shows that the mean years of education has a maximum of only 8.2 years with the overall average of only 3.7 (Table 1). Furthermore, men received more than twice as many years of education on average as compared to women. The general low level of education attainment in the region is further evidenced by the highest percentage of men aged 20–49 who work in agriculture related jobs, which do not require schooling. With a variance of 11, the wealth index indicates the disparity of wealth that exists in these countries (Table 1). The relatively poor sanitation conditions of these countries are evident in the fact that few households have a toilet or even piped water. Instead, many practice open defecation where *in lieu* of modern facilities, individuals use fields, bushes, forests, or bodies of water to defecate. Finally, the stark contrast in urban versus rural areas is captured by the extreme values and high standard deviation shown by population density (Table 1).

Country-specific summary statistics are shown in Table 4. On average, Sierra Leone has the highest percentage of individuals working in agricultural jobs. Guinea appears to be a more developed country as it boasts a higher percentage of households with telephone, television, piped water, high quality floors, and flush toilets. Each country has both rural and metropolitan areas as evidenced by the variation in population density, with Liberia displaying a lower overall density when compared to Guinea and Sierra Leone.

**Table 3:** Distribution of population, total number of confirmed cases, and risk of EVD by country affected.

**Country**	**Population**	**Confirmed cases of Ebola**	**Risk (cases/100,000)**
Guinea	10,628,972	3,335	31
Liberia	3,476,608	3,151	91
Sierra Leone	6,079,086	8,683	143
**Total**	**20,184,666**	**15,169**	**75**

### Poisson and negative binomial models

Results from univariable and multivariable Poisson models are shown in Tables 5 and 6, respectively. There is evidence of overdispersion in the multivariable Poisson model with $ \frac{1}{df} \mathrm{Deviance}=138$. Therefore, a negative binomial model was fit to the data to assess the association between the outcome and the predictor variables. All subsequent discussions are based on the negative binomial model.

**Table 4:** Summary statistics for the variables obtained for use in the model summarized by country.

**Variable**	**Min**	**Max**	**Mean**	**Standard deviation**
**Country: Guinea**				
Wealth index	26.2	72.6	35.4	15.5
Mean years of education persons 20–49	1.5	7.3	2.9	1.9
Mean years of education of women 20–49	0.7	5.7	1.8	1.6
Mean years of education of men 20–49	2.4	9	4.2	2.1
Percent population living in urban area	5.4	100	27.5	30.6
Percent married men age 20–49 working in agriculture	3.7	76.4	56	23.7
Percent married men age 20–49 working in blue collar jobs	20.6	73.2	37.5	18.1
Percent married men age 20–49 working in white collar jobs	1.8	23.1	6.4	6.9
Percent of households with a television	7.3	85.2	23.4	26
Percent of households with a telephone	48.5	97.6	66.9	14.2
Percent of households with electricity	4.9	94	22.2	29.9
Percent of households with 0–1 rooms designated for sleeping	6.1	15.5	10	3.6
Percent of households with >3 rooms designated for sleeping	56.5	74.3	66.5	8.3
Percent of households with a high quality floor	1.3	33	7	10.7
Percent of households with a low quality floor	5.1	66.4	46.5	18.6
Percent of households with piped water	2	84.7	17.6	28.2
Percent of households with bad quality water supply	0.9	49.2	28.2	16.4
Percent of households with a flush toilet	1.9	62.5	15.4	20.3
Percent of households with bad quality or no toilet	8.9	74.9	59.2	21
Population density	26.5	3,706.4	497.5	1,296.6
**Country: Liberia**				
Wealth index	15.3	45.9	23.5	7.5
Mean years of education persons 20–49	3.3	8.2	4.7	1.3
Mean years of education of women 20–49	1.6	6.9	3	1.4
Mean years of education of men 20–49	4.9	9.5	6.4	1.2
Percent population living in urban area	4.4	93.4	33.5	24.1
Percent married men age 20–49 working in agriculture	12.3	75.3	49.6	14.9
Percent married men age 20–49 working in blue collar jobs	15.6	71.7	41.4	13.2
Percent married men age 20–49 working in white collar jobs	4.9	16	8.9	2.9
Percent of households with a television	0.7	39	7.5	9.7
Percent of households with a telephone	34.7	92.3	54.6	14.9
Percent of households with electricity	0	26.2	4	6.5
Percent of households with 0–1 rooms designated for sleeping	8.8	35.8	20.3	7.5
Percent of households with >3 rooms designated for sleeping	30.6	70.9	50.5	11.2
Percent of households with a high quality floor	0.4	19.7	3.4	4.9
Percent of households with a low quality floor	13	92.4	64.9	20
Percent of households with piped water	0	10.5	0.8	2.7
Percent of households with bad quality water supply	12.3	75.9	35.7	19.4
Percent of households with a flush toilet	0.1	42	7	11.9
Percent of households with bad quality or no toilet	31.7	89.6	68	14.3
Population density	8.4	594.8	67.2	147.3
**Country: Sierra Leone**				
Wealth index	16.2	50.7	23.9	8.8
Mean years of education persons 20–49	1.6	7.3	3	1.4
Mean years of education of women 20–49	1	6.1	2.1	1.3
Mean years of education of men 20–49	2.4	8.5	4	1.6
Percent population living in urban area	5.7	91.9	23.8	22.5
Percent married men age 20–49 working in agriculture	10.5	84	64.6	18.7
Percent married men age 20–49 working in blue collar jobs	10.5	72.7	29	15.7
Percent married men age 20–49 working in white collar jobs	2.6	16.8	6.4	3.7
Percent of households with a television	2.2	55.7	9.4	14.5
Percent of households with a telephone	28.8	91.5	53.9	15
Percent of households with electricity	0.5	58.2	8.3	16.1
Percent of households with 0–1 rooms designated for sleeping	4.5	29.6	13.4	5.8
Percent of households with >3 rooms designated for sleeping	39.7	77	60.9	8.6
Percent of households with a high quality floor	0.7	23.4	3.5	6.1
Percent of households with a low quality floor	10.3	81.5	64.7	18.2
Percent of households with piped water	0.1	24.7	2.8	6.6
Percent of households with bad quality water supply	9.3	79.1	46.9	21.8
Percent of households with a flush toilet	0.3	21	2.8	5.6
Percent of households with bad quality or no toilet	20.1	81.7	55.3	16.5
Population density	20.7	3,014.8	295.3	817.6

**Table 5:** Results of univariable Poisson models showing unadjusted associations between suspected predictors and Ebola Virus Disease risk.

	**Unadjusted**			
**Variable**	** Risk Ratio**	**95% C.I.**	**SE**	***p*-value**
Wealth index	1.006	(1.005, 1.007)	0.0005	<0.001
Mean years of education persons 20–49	1.203	(1.195, 1.210)	0.004	<0.001
Mean years of education of women 20–49	1.227	(1.218, 1.235)	0.004	<0.001
Mean years of education of men 20–49	1.186	(1.178, 1.193 )	0.004	<0.001
Percent population living in urban area	1.009	(1.009, 1.009)	0.0002	<0.001
Percent married men age 20–49 working in agriculture	0.989	(0.988, 0.990)	0.0003	<0.001
Percent married men age 20–49 working in blue collar jobs	1.013	(1.012, 1.013)	0.0004	<0.001
Percent married men age 20–49 working in white collar jobs	1.049	(1.049, 1.051)	0.001	<0.001
Percent of households with a television	1.005	(1.0047, 1.006)	0.0003	<0.001
Percent of households with a telephone	1.013	(1.012, 1.014)	0.0005	<0.001
Percent of households with electricity	1.004	(1.003, 1.005)	0.0003	<0.001
Percent of households with 0–1 rooms designated for sleeping	1.049	(1.047, 1.051)	0.0009	<0.001
Percent of households with >3 rooms designated for sleeping	0.967	(0.966, 0.968)	0.0006	<0.001
Percent of households with a high quality floor	1.023	(1.021, 1.024)	0.0007	<0.001
Percent of households with a low quality floor	0.989	(0.988, 0.990)	0.0003	<0.001
Percent of households with piped water	0.996	(0.995, 0.997)	0.0004	<0.001
Percent of households with bad quality water supply	0.999	(0.998, 1.0)	0.0004	0.124
Percent of households with a flush toilet	1.004	(1.003, 1.004)	0.0004	<0.001
Percent of households with bad quality or no toilet	0.983	(0.982, 0.983)	0.0003	<0.001
Population density	1.0	(1.0, 1.0)	<0.00001	<0.001

**Table 6:** Results of final Poisson model showing significant predictors of confirmed cases of Ebola Virus Disease in West Africa.

	**Adjusted**			
**Variable**	**Risk Ratio**	**95% CI**	**SE**	***p*-value**
Wealth index	0.707	(0.682, 0.733)	0.013	<0.001
Mean years of education persons 20–49	0.005	(0.003, 0.009)	0.002	<0.001
Mean years of education of women 20–49	28.1	(20.1, 39.2)	4.78	<0.001
Mean years of education of men 20–49	15.5	(11.6, 20.7)	2.30	<0.001
Percent population living in urban area	0.973	(0.969, 0.977)	0.002	<0.001
Percent married men age 20–49 working in agriculture	3.43 × 10^−8^	(9.86 × 10^−9^, 1.19 × 10^−7^)	2.18 × 10^−8^	<0.001
Percent married men age 20–49 working in blue collar jobs	3.19 × 10^−8^	(9.14 × 10^−9^, 1.11 × 10^−7^)	2.03 × 10^−8^	<0.001
Percent married men age 20–49 working in white collar jobs	3.60 × 10^−8^	(1.04 × 10^−8^, 1.25 × 10^−7^)	2.28 × 10^−8^	<0.001
Percent of households with a telephone	1.063	(1.055, 1.070)	0.004	<0.001
Percent of households with electricity	1.013	(1.004, 1.023)	0.005	0.004
Percent of households with 0–1 rooms designated for sleeping	0.852	(0.841, 0.863)	0.006	<0.001
Percent of households with >3 rooms designated for sleeping	0.925	(0.917, 0.932)	0.004	<0.001
Percent of households with a high quality floor	1.610	(1.555, 1.658)	0.026	<0.001
Percent of households with a low quality floor	0.992	(0.986,0.998)	0.003	0.007
Percent of households with piped water	1.009	(0.999, 1.018)	0.005	0.05
Percent of households with bad quality water supply	0.969	(0.966, 0.973)	0.002	<0.001
Percent of households with a flush toilet	0.957	(0.945, 0.969)	0.006	<0.001
Percent of households with bad quality or no toilet	0.968	(0.964, 0.972)	0.002	<0.001
Population density	0.999	(0.999, 0.999)	0.00008	<0.001

Results from univariable and multivariable negative binomial models are shown in Tables 7 and 8, respectively. Mean years of education of persons 20–49, percent of population living in urban areas, percent of households with bad quality or no toilets, and percent of married men aged 20–49 working in blue collar jobs were significantly associated with the risk of EVD. Results from the multivariable negative binomial model indicated that regions with higher percentages of: (a) the population living in urban areas, (b) households with bad quality or no toilet, and (c) married men age 20–49 working in blue collar jobs tended to have lower risks of EVD whereas those with higher average education level tended to have significantly higher risks of EVD.

**Table 7:** Results of univariable negative binomial models showing unadjusted association assessments between Ebola Virus Disease and each of the potential predictors investigated.

**Variable**	**Unadjustedrisk ratio**	**95% C.I.**	**SE**	***p*-value**
Wealth index	1.01	(0.980, 1.05)	0.017	0.422
Mean years of education persons 20–49	1.15	(0.931, 1.43)	0.125	0.194
Mean years of education of women 20–49	1.20	(0.936, 1.55)	0.154	0.149
Mean years of education of men 20–49	1.13	(0.933, 1.37)	0.110	0.212
Percent population living in urban area	1.01	(0.994, 1.02)	0.007	0.30
Percent married men age 20–49 working in agriculture	0.995	(0.980, 1.01)	0.008	0.550
Percent married men age 20–49 working in blue collar jobs	1.0	(0.985, 1.02)	0.009	0.720
Percent married men age 20–49 working in white collar jobs	1.07	(0.980, 1.18)	0.050	0.128
Percent of households with a television	1.01	(0.987, 1.03)	0.011	0.442
Percent of households with a telephone	1.01	(0.993, 1.04)	0.011	0.195
Percent of households with electricity	1.01	(0.986, 1.03)	0.010	0.521
Percent of households with 0–1 rooms designated for sleeping	1.01	(0.967, 1.05)	0.022	0.683
Percent of households with >3 rooms designated for sleeping	0.989	(0.962, 1.02)	0.014	0.412
Percent of households with a high quality floor	1.03	(0.977, 1.09)	0.028	0.268
Percent of households with a low quality floor	0.989	(0.972, 1.01)	0.008	0.191
Percent of households with piped water	0.997	(0.972, 1.02)	0.013	0.826
Percent of households with bad quality water supply	0.998	(0.982, 1.01)	0.008	0.839
Percent of households with a flush toilet	1.01	(0.984, 1.04)	0.014	0.430
Percent of households with bad quality or no toilet	0.974	(0.952, 0.995)	0.011	0.018
Population density	1.0	(1.0, 1.0)	0.0002	0.370

**Table 8:** Results of final negative binomial model showing significant predictors of Ebola Virus Disease risk in West Africa.

**Variable**	**Adjusted risk ratios**	**95% CI**	**SE**	***p*-value**
Mean years of education of persons 20–49	2.27	(1.29, 3.99)	0.652	0.004
Percent population living in urban areas	0.95	(0.922, 0.987)	0.017	0.006
Percent of households with bad quality or	0.95	(0.923, 0.979)	0.015	0.001
no toilet				
Percent of married men age 20–49 working	0.96	(0.928, 0.996)	0.017	0.027
in blue collar jobs				

**Figure 2:**
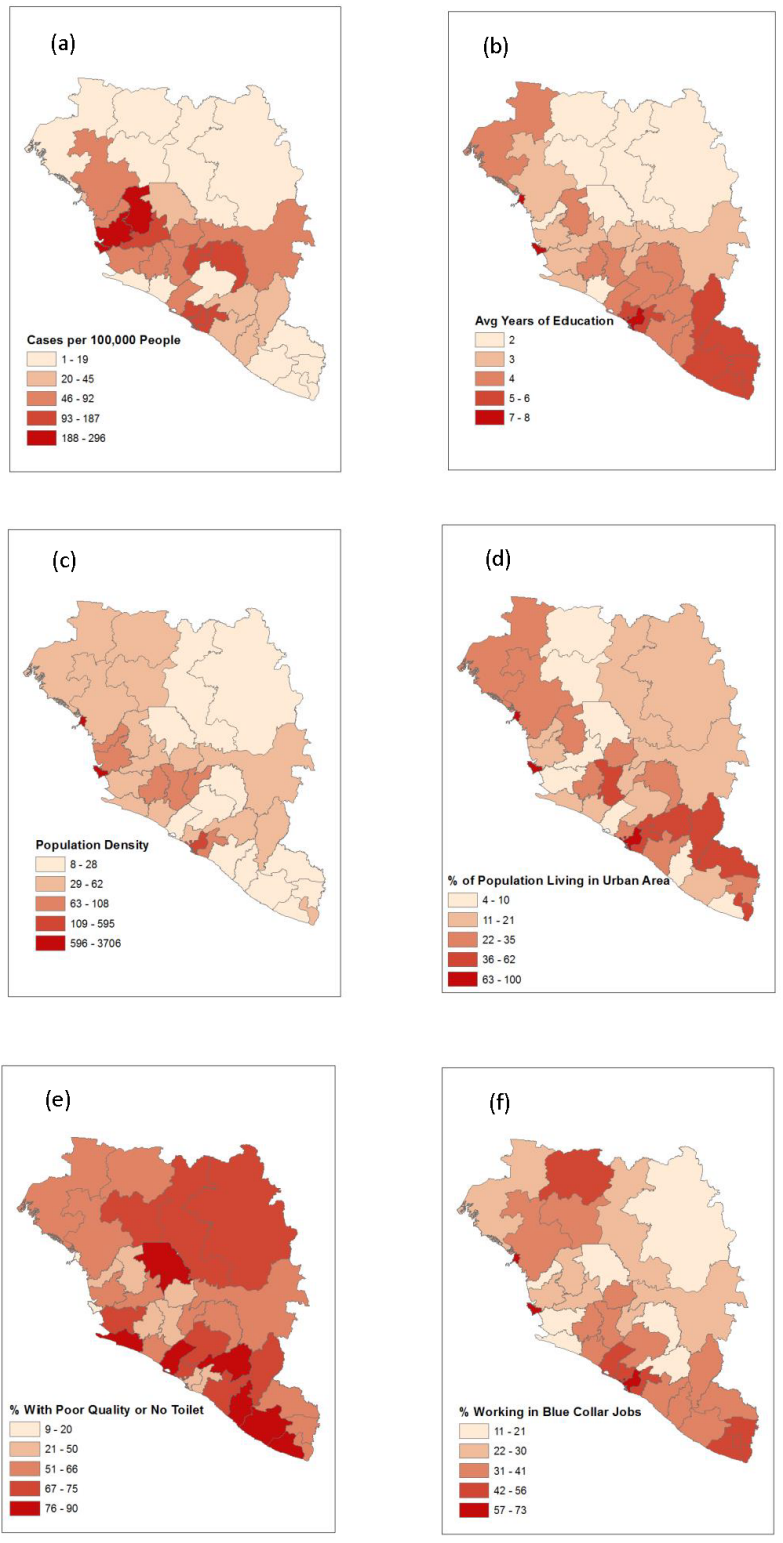
A map depicting the outcome of interest, all four significant predictor variables, and density of individuals. (A) Risk of EVD. (B) Average years of education. (C) Population density. (D) Percent of population living in an urban area. (E) Percent of population with a poor quality or no toilet. (F) Percent of male population that works in a blue collar job.

The four predictors found to be significant in the multivariable negative binomial model, and density of individuals, are compared in Fig. 2. Something that becomes evident in Fig. 2E is the large number of regions in which most individuals do not have access to a quality toilet. Locations with a high percentage of men who work in blue collar jobs appear to coincide with those that have high population density in a given region. Comparing Figs. 2B and 2D, it seems that locations with a higher average level of education tended to have a high percentage of the population residing in urban areas. However, locations that had a high percentage of the population living in urban areas did not necessarily have high population density (Figs. 2C and 2D). Some locations have a high level of urbanization and low population density which implies that the region’s population is concentrated in several cities with few individuals living in rural areas; Grand Gedeh is an example of such a region (Figs. 1, 2C and 2D) .

## Discussion

This study investigated region level predictors of Ebola risk in the three West African countries of Guinea, Liberia, and Sierra Leone. Most similar to this study are studies by Fang and Valeri (Fang et al., 2016; Valeri et al., 2016). All three studies were conducted at the same spatial scale and relate similar predictor variables to slightly different outcome variables related to EVD during the 2014 outbreak. While our study ultimately relied on results from a negative binomial model, the study by Fang and co-workers used a Poisson model to analyze associations, and Valeri and co-workers used multivariable linear regression models. A key difference in all three models is how the outcome of interest was measured. In the study by Fang et al. the authors used transmissibility as their dependent variable, which is defined as the average number of secondary infections caused by a patient per week, whereas Valeri and co-workers compared predictors to the final epidemic size and final epidemic proportion. In our study, we used disease risk as the outcome and investigated population (region level) predictors of the outcome. In some cases our findings agree with those of previous studies. For instance, regions with high average education level tended to have a high risk of EVD (Valeri et al., 2016). Other times differences in model structure and/or measurement of the outcome of interest resulted in conflicting results. For example, while Fang and co-workers (2016) showed a significant positive relationship between population density and increased transmission of the disease in Sierra Leone, Valeri et al. (2016) and our study did not find a significant relationship between population density and risk/epidemic size of EVD.

One common theme illustrated by several studies is how rural locations play a significant yet unclear role in large scale outbreaks of EVD. The spread of Ebola is driven by known risk factors including caring for sick neighbors and key gatherings such as marriages and funerals (Brainard et al., 2016; Reza et al., 2015). Local community effects are known to have been important in the 2014 epidemic (Faye et al., 2015; Nyenswah et al., 2015) and the social ties that necessitate these events are particularly strong in rural settings (Richards et al., 2015). Additionally, incidents can be concentrated along roads connecting rural towns to cities (Lu et al., 2015), which is substantiated by a separate study that shows how those traveling long distances are more likely to be contributing to the spread of the disease (Agua-Agum et al., 2016). Pair this information with the likely existence of superspreaders in the population (Agua-Agum et al., 2016; Lau et al., 2017; Richards et al., 2015) and the fact that EVD antibody prevalence is highest in rural areas (Moyen et al., 2015), we begin to see how rural locations are important in outbreaks of the disease. Epidemic responses tend to focus on locations with large populations because high population densities are typically associated with higher cumulative case counts of EVD (Fang et al., 2016). However, strong social ties often bring a large number of rural-dwelling people together from long distances creating a poorly understood harbor for the disease and an ideal scenario for a potential superspreader (Agua-Agum et al., 2016; Brainard et al., 2016; Lau et al., 2017). Since rural locations link urban areas, contain complex social networks and are difficult to monitor, they likely play a larger role in outbreaks than we currently understand.

The relationship between risk of EVD with population density and the proximity to rural areas is not well understood. To highlight this fact further, consider the study by (Walsh & Haseeb, 2015), which reports a positive association between zoonotic transmission of EVD and increased vegetation in areas with high population density and a negative association in areas with low population density. One explanation for the inconsistencies could be that since the study by Fang et al. (2016) only considered Sierra Leone while this study and Valeri’s study (Valeri et al., 2016) also included Guinea and Liberia, population density may have played a larger role at the region level in Sierra Leone specifically. The disparity between the size of regions in the three countries may also be related to population density failing to be a significant predictor at the spatial scale at which this study and Valeri’s study were conducted. Specifically, it is unlikely that population density is homogeneous throughout the comparatively large regions in Guinea whereas the more compact regions in Sierra Leone are likely more homogeneous, resulting in their population density values representing a more accurate indicator of the concentration of individuals throughout the entire area. This is clear when viewing Fig. 2 as one will notice that many locations exhibiting a high percentage of the population living in an urban area correspond to regions with low overall population density. The inconsistent relationship between population density and risk of EVD paired with the unclear, but significant, role rural regions play in harboring the disease in an outbreak hinder our ability to make causal inferences.

It has been shown that EVD spreads between urban areas with high population density via rural locations that connect them (Szendroi & Gábor, 2004; Lu et al., 2015). In our study area, many regions with high percentages of the population living in an urban area tended to be regions with low overall population density. This implies that in such regions much of the population resides in a small number of urban cities and that the remaining rural locations are very sparsely populated. Since there was a lower rural influence on the spread of disease in these regions, EVD was less likely to spread spatially across the landscape connecting areas of high population. Additionally, overseeing health care needs during an outbreak in an urban setting is more manageable since there are fewer physical locations that decision makers would be concerned with; health interventions can be concentrated in a smaller number of locations and, in the absence of rural influence on the spread and harboring of the Ebola virus, more easily contain the disease (Agua-Agum et al., 2016). For example, Grand Gedeh and Grand Cape Mount in Liberia have similar population densities, but Grand Cape Mount has a much lower percent of population living in urban areas. These conditions allowed the disease to more easily spread spatially between urban locations through the more densely populated rural areas in Grand Cape Mount, which contributed to a significantly higher disease risk.

The geographic distribution of average level of education was similar to that of Ebola risk per 100,000 individuals, except in the southeastern regions where EVD risk was much lower (Fig. 2). While the southeastern regions are among the most educated, they also had the highest population living in an urban area, highest percentage of workers in blue collar jobs, and a large percentage of the population with poor quality toilets, all of which were negatively associated with EVD risk. Sierra Leone has a relatively high average level of education, low percentage of the population living in an urban area, low percentage of individuals with blue collar jobs, and a low percentage of individuals with a poor quality or no toilet. These factors contributed to Sierra Leone having the highest risk per 100,000 individuals as seen in Table 3.

More urbanized areas have improved infrastructure and therefore better access to modern toilets, and residents have increased access to schools which leads to a higher overall education level (Valeri et al., 2016). Local industry and employment opportunities are also related to the level of urbanization in a given region. Male employment is categorized in three ways in our dataset: those related to agriculture, upper-level professional positions, and entry-level jobs that are not related to agricultural and do not require an education. Professional occupations require a certain level of education such as managerial and technical jobs while entry-level positions include manual labor, clerical positions, sales positions and other “blue collar” jobs. While agricultural jobs are more prominent in rural locations, there are more professional and entry-level positions in cities. Increased urbanization is therefore associated with each of the significant variables in our multivariable negative binomial model. However, regions with higher average education level were associated with higher risks of EVD in our model while the other predictors were negatively associated with Ebola risk. These results may indicate that the percentage of individuals living in an urban area, average education level, percent of households without a flush toilet, and percent of men with blue collar jobs may be proxy measures for other factors in urban areas. This reiterates what several studies have already stressed, that there is a great need to better understand the unique nature of social and work-related interactions in rural areas, especially as they relate to urban areas and the existence of superspreaders (Brainard et al., 2016; Richards et al., 2015).

The well-established mode of transmission for Ebola is contact with bodily fluids of an infected individual (Reza et al., 2015; World Health Organization, 2015). There is also agreement among studies at the individual level about contact-related risk factors that increase the likelihood of contracting and spreading EVD. These risks include direct care for individuals with the disease, traveling long distances, and attending funerals of those who have died of Ebola (Agua-Agum et al., 2016; Brainard et al., 2016; Francesconi et al., 2003; Victory et al., 2015). These activities are deeply rooted in cultural practices and are important parts of West African culture. Several of these activities, such as caring for sick individuals and attending funerals, are especially important and therefore pronounced in rural communities, further emphasizing the need to study these areas in more depth (Lau et al., 2017; Richards et al., 2015).

Regions with higher levels of urbanization were associated with lower risk of EVD probably because of improved access to health care facilities and decreased individual travel, each of which have been shown to protect against the disease (Agua-Agum et al., 2016; Brainard et al., 2016). Furthermore, an overlooked negative side effect of the presence of flush toilets is the creation of a commonly visited place where bodily fluids from numerous individuals are concentrated. Locations that exhibit a high percentage of bad quality or no toilet may imply that a large portion of people in the region practice open defecation, where individuals defecate in fields, forests, or other open spaces rather than using a common toilet. Thus, while improved access to flush toilets in urban areas has many benefits, it is possible it also increases opportunities of contact with bodily fluids of infected individuals, especially if good hygienic practices such as proper hand washing and proper cleaning of toilets are not well practiced.

More comprehensive and finer-scale data would improve future studies. For instance, our three occupational categories only include data on men and since Valeri found that the percentage of women in a population also acts as a risk factor, more data related to women in the work force is needed (Valeri et al., 2016). Average education level being associated with higher risk of EVD is corroborated by (Valeri et al., 2016) using a linear regression at the same spatial scale and spatial extent. Improved data may help explain this relationship. In the absence of such information we reiterate that education level is likely a proxy for an unmeasured behavior or relationship. For example, one possibility that would explain our results related to average education would be if the more educated individuals or regions have better access to accurate EVD testing, records of which are more likely to be reported and available. This is a plausible explanation as data related to the 2014 EVD outbreak in West Africa is generally limited and can be unreliable. Improved information related to the contact structure in rural locations related to harboring and spreading the disease as well as the existence of superspreaders would also aid interpretation of these and other results, a fact echoed by several studies (Brainard et al., 2016; Richards et al., 2015; Walsh & Haseeb, 2015). In designing future studies it is important to collect data over a consistent spatial scale since spatial inconsistencies may mask relationships and hinder a thorough analysis, which is evident in this and other studies (Fang et al., 2016; Lau et al., 2017; Valeri et al., 2016).

## Conclusions

Our results indicate that mean years of education of persons age 20–49 is associated with higher EVD risk. This finding is confirmed by a related study and calls for additional research into the relationship (Valeri et al., 2016). We found that percentage of population living in urban areas, percentage of households with bad quality or no toilets, and percentage of married men aged 20–49 working in blue collar jobs were significantly associated with lower risk of EVD. All of our results emphasize the relevance of urbanization. Our findings also suggest that the relationship between Ebola and population density requires more research. Additional and improved data allowing more fine-scale and spatially consistent research would help resolve some of these issues and provide clarity in interpreting results.

##  Supplemental Information

10.7717/peerj.5888/supp-1 Supplemental Information 1 Ebola STATA code

10.7717/peerj.5888/supp-2 Supplemental Information 2 STATA file containing predictor variables, population values, and case counts used in our analysis

10.7717/peerj.5888/supp-3 Supplemental Information 3 Excel file containing predictor variables, population values, and case counts used in our analysis

## Additional Information and Declarations

### Data Availability

The following information was supplied regarding data availability: The raw data and STATA code are available in the Supplemental Files.
